# This needs to be a journey that we’re actually on together’—the introduction of integrated care systems for children and young people in England: a qualitative study of the views of local system stakeholders during winter 2021/22

**DOI:** 10.1186/s12913-023-10442-6

**Published:** 2023-12-20

**Authors:** Steven Hope, Evgenia Stepanova, Oliver Lloyd-Houldey, Frances Hillier-Brown, Dougal Hargreaves, Dasha Nicholls, Carolyn Summerbell, Russell M. Viner, Zainab Dedat, Emily C. Owen, Stephanie Scott

**Affiliations:** 1 Population, Policy and Practice Research and Teaching Department, UCL Great Ormond Street Institute of Child Health, UCL, London, UK; 2 Population Health Sciences Institute, Newcastle University, Newcastle Upon Tyne, UK; 3 Department of Sport and Exercise Sciences, Durham University, Durham, UK; 4 Fuse – Centre for Translational Research in Public Health, Newcastle Upon Tyne, UK; 5 Mohn Centre for Children’s Health & Wellbeing, School of Public Health, Imperial College London, London, UK; 6 Division of Psychiatry, Department of Brain Sciences, Imperial College London, London, UK; 7 East London NHS Foundation Trust, London, UK; 8 Department of Primary Care & Population Health, UCL, London, UK

**Keywords:** Integrated care, Child health, Health policy, Paediatrics, Health systems

## Abstract

**Background:**

Integrated care has become a central feature of health system reform worldwide. In England, Integrated Care Systems (ICS) are intended to improve integration across public health, the National Health Service (NHS), education and social care. By April 2021, England had been divided into 42 geographical areas, each tasked with developing local ICS provision*.* However, it was not clear how ICSs would address the specific needs of children and young people (CYP). This study elicited the views of senior professional stakeholders in the first year of the ICS national roll out, to learn how integrated care for CYP was being implemented within the ICSs and future plans for service provision.

**Methods:**

A qualitative analysis of in-depth interviews with stakeholders, including healthcare professionals, NHS managers and local authority leaders (*n* = 25) selected from a diverse sample of ICSs (*n* = 7) across England, conducted during winter 2021/22. Reflexive thematic analysis involving a collaborative coding approach was used to analyse interview transcripts.

**Results:**

Four themes were identified, indicating challenges and opportunities for ICSs in relation to the health of CYP: 1) Best start in life (a more holistic approach to health afforded by integrated care); 2) Local and national contexts (tensions between local and national settings and priorities); 3) Funding and planning (instituting innovative, long-term plans using limited existing CYP funding streams); 4) Organisational complexities (integrating the work of diverse organisations).

**Conclusions:**

The views of stakeholders, provided at the beginning of the journey towards developing local ICS CYP provision, revealed a common aspiration to change focus from provision of acute, largely adult-orientated services towards one with a broader, population health remit, including prevention and early intervention. This would be delivered by integration of a range of local services, including health, education, housing and social care, to set CYP on a life-long path towards improved health and wellbeing. Yet there was an awareness that change would take place over time within existing national policy and funding frameworks, and would require overcoming organisational barriers through further developing local collaborations and partnerships. As ICSs mature, the experiences of stakeholders should continue to be canvassed to identify practical lessons for successful CYP integrated care.

**Supplementary Information:**

The online version contains supplementary material available at 10.1186/s12913-023-10442-6.

## Background

Integrated care has become a central feature of health system reform worldwide, with the aim of tackling the impact of increased demand and improving care through reducing the fragmentation of services [1, 2]. The World Health Organization (WHO) framework on integrated care proposes that a move towards greater integration provides opportunities to strengthen governance and co-ordination of services, empower individuals and communities, and improve the quality and efficiency of healthcare and population health [3]. Joining up pathways of care, through integrated care models, has been argued as an essential goal for those who regulate, deliver and receive care worldwide [4].

However, models of integration have been largely designed around the health and service needs of adults rather than those of children and young people (CYP), and while integrated care has been linked to better outcomes for adults there is limited evidence for the success of integrated care for CYP [5]. There are distinct characteristics of integrated care for CYP, due to the significant roles of family and education, and changing needs across childhood and adolescence. For CYP, integrated care will encompass vertical integration (between primary and secondary care), horizontal integration (between health, education and social sectors), longitudinal integration (developmentally appropriate co-ordination of health and non-health services), and population integration (taking a public health focus, including health promotion strategies and preventative measures alongside clinical care) [6, 7].

While systems of integrated care for CYP have been developed around the world, replication can be challenging due to variations in national, local and health service contexts, together with differences in integrated care provision for specific health conditions [8]. In England, there have been many examples since the 1950s of attempts to increase integration of CYP services within the National Health Service (NHS), with the ambition to develop better and more efficient planning, coordination, decision-making and prioritisation of health and care needs across a range of services [9]. Nevertheless, integrated care has progressed “in fits and starts” [9], with a lack of evidence for the generalisability and transferability of earlier examples of integrated care in England [7].

Against this backdrop of a limited evidence base and repeated earlier attempts to improve CYP integrated care, the 2019 NHS Long-Term Plan in England [10] set out a new vision for the NHS, including action to better support CYP health and wellbeing. One of the key proposals of the Long-Term Plan was the introduction across England of Integrated Care Systems (ICSs). An ICS comprises a local footprint responsible for integration of services within a geographical area. ICSs adopt and extend the functions and statutory responsibilities previously held by Clinical Commissioning Groups (CCGs), including planning, commissioning and co-ordinating services in a geographical footprint, although for larger areas than those represented by CCGs, and with a greater focus on population and system-level planning [11]. ICSs are also tasked with adopting Special Educational Needs and Disabilities (SEND) and safeguarding duties that had been held by CCGs, and have statutory responsibilities to ensure that constituent organisations within the ICS comply with the safeguarding obligations for CYP.

The NHS Long-Term Plan specifically introduced a new system focus for CYP, supporting strategic planning of healthcare across the NHS, public health and other services, such as education and social care. From a population health perspective, core purposes assigned to ICSs include the improvement of outcomes in population health and healthcare, alongside tackling inequalities in outcomes, experience and access in local areas [12]. ICSs were rolled out in early 2021, with England served by 42 geographical ICS footprints. While the broad remit of ICS provision is established by statute and overseen nationally by NHS England, a principal aim of this policy is to promote flexibility, with ICSs granted a large degree of autonomy in how they plan and carry out work within their local context, taking into account differences according to need, as well as existing services and integration experiences.

The qualitative study described in this paper was carried out as part of a larger project within the NIHR School of Public Health Research that used a variety of research approaches to explore how developing ICSs in England considered the needs of CYP, to review measurement instruments for integration [13], and to understand key components of integrated care for this age group [14]. We sought to canvas the views and experiences of key stakeholders (i.e. senior, experienced members of staff in clinical or managerial roles with an important perspective on ICS CYP provision, including healthcare professionals, NHS managers and local authority leaders) in a diverse sample of ICSs in England during the first year of the national rollout, to learn how integrated care for CYP was being implemented locally and to gain insights into the future plans of ICSs.

## Methods

### Study context and design

A qualitative interview study was undertaken in a diverse sample of ICSs in England between November 2021 and March 2022. In-depth, semi-structured online interviews were carried out with three or more key clinical or managerial stakeholders from each sampled ICS. The context for the study is important because it provides the background to the data collection. Two key contextual factors are relevant. First, as stated in the introduction, interviews were conducted during the first year of national ICS rollout. Second, the interviews (and ICS rollout) occurred during the COVID-19 pandemic, a significant stressor facing the NHS [15]. The pandemic not only increased demands for services but also required ICSs to rapidly introduce innovative working practices [16].

Responding to the pandemic provided unavoidable context to participant accounts rather than representing a demonstrable theme relating to the development of ICSs. The impact of the pandemic was felt by interviewees from all ICSs. For example, resources had been moved from CYP to adult provision, and stakeholders reflected that CYP had been affected directly and indirectly by the pandemic through changes to service delivery. Further, the impact of the pandemic on inequality and CYPs’ mental health was raised by stakeholders in all ICSs as an area of particular concern. Nevertheless, the pandemic had positive repercussions, such as the acceleration of partnership working.

### Recruitment and sample

#### ICS recruitment

We constructed a sampling frame comprising 38 of the 42 ICSs (91%) in England who had provided data to us in an online survey conducted during an earlier stage of the project [17]. This sampling frame was chosen due to availability of pre-interview data. We then used purposive sampling to identify 10 ICSs for inclusion in the qualitative study according to three characteristics: maturity of the CYP system (derived from answers to a question in the online survey about perceived progress towards being a fully functional ICS for CYP, on a scale of 1–5 [recoded: low (1–2), medium (3) and high (4–5)]); deprivation (based on Indices of Multiple Deprivation 2019 scores relating to each ICS area, coded as high, medium, or low level); and rurality (whether the ICS area was predominantly rural or urban). The selected ICSs were geographically spread across England.

An email was sent to the CYP services contact person in each ICS (identified from publicly available information supplemented with details provided by the NHS England CYP Programme and NHS England regional leaders), asking them to identify 3–4 key stakeholders within the ICS for interview. A follow up email was sent after seven days if there had been no response. Seven ICSs were recruited in total. The remaining ICSs approached were unable to take part due to capacity issues during the fieldwork period, which coincided with a surge in COVID-19 infections and a related vaccination drive. We attempted to replace these ICSs with others that shared key characteristics. However, none of the replacements were able to take part due to similar capacity constraints.

#### Stakeholder recruitment

Sampled ICSs were asked to nominate key stakeholders within the local ICS footprint to be interviewed. We defined a ‘key stakeholder’ to be a senior, experienced member of staff in a clinical or managerial role, with an important perspective on ICS CYP provision. ICSs were provided with a list of potential stakeholder roles they might consider, including: clinical CYP lead; non-clinical CYP lead; operational CYP lead; regional lead; key decision-makers (e.g. a Director of Children’s services); a local authority representative. The research team contacted each selected stakeholder using an email address provided by the ICS, inviting them to take part in an interview. If a selected stakeholder could not take part, the ICS was asked to provide the contact details of an alternative stakeholder who was then contacted by email. 33 stakeholders were contacted in total, of whom 25 agreed to be interviewed (76% of the stakeholders contacted). An information sheet and a consent form were sent to stakeholders in advance, and consent was verbally re-established before the interview took place.

### Data collection

Interviews were guided by a schedule developed from an a-priori list of CYP integrated care topics generated by the project team; this schedule is included in the supplementary material (see Additional file 1). Topic areas and questions were refined in five pilot interviews with policy and practice professionals who had expertise in integrated care in England, and in an engagement session with young people and their parents/caregivers. Interview schedule topics included: ICS programme and priorities; Partnerships, resources, and capacity; Leadership; Information sharing mechanisms; Engagement and empowerment of service users; Measuring integration; COVID-19 impacts; and Future plans. Interviews were conducted online using MS Teams and averaged 45 to 60 min.

### Analysis

All interviews were video-recorded and transcribed verbatim by a professional transcription company (“Way with Words”) with observational fieldnotes maintained in a research diary. Interview transcripts were analysed using reflexive thematic analysis [18]. In this analysis, we used a collaborative coding approach designed to develop a richer, more nuanced reading of the data, rather than simply to seek consensus on meaning [19]. The raw transcript text of each interview was first coded line-by-line by one of six members of the research team [ES, SH, SS, OL-H, ZD, EO]. We began with an a-priori list of thematic codes based on the interview schedule topics, which were supplemented iteratively by codes identified during familiarisation and the first stage of the coding process. Codes were then systematically indexed into data tables to generate detailed descriptive themes.

The research team critically discussed and challenged these descriptive themes at regular analysis meetings, using a process known as pragmatic double-coding [20]. Descriptive themes were compared to identify patterns, similarities and differences in the data, and relationships between them were elaborated in order to generate analytical themes and a consistent interpretation of the dataset as a whole. Finally, we were mindful that stakeholder accounts were nested within a particular ICS. Therefore, in addition to the analysis of individual transcripts, we explored similarities and differences in the accounts from interviewees from the same ICS, and wrote narrative case notes which we also discussed and challenged as a research team. We used these narrative case notes to compare and contrast against the wider descriptive themes, adapting and amending our thematic structure to ensure themes were comprehensive of findings across all ICSs.

Several established approaches were taken to ensure the validity and rigour of the findings including development of a collaborative coding system, peer review of themes, and provision of thick description that recognises the context of data collection, supported by illustrative quotes and detailed field notes [21].

## Results

Participating ICSs varied by maturity, deprivation and rurality (Table 1). In terms of progress towards becoming a ‘fully functional ICS for CYP’, three of the ICSs had previously rated themselves high maturity and four medium maturity; no sampled ICS rated low maturity was able to participate due to reduced staff capacity during the pandemic. Twenty-five stakeholder interviews were conducted, comprising 3–5 interviewees from each ICS. Twenty of the stakeholders were based in the NHS while five were based in local authorities, although some participants currently working within the NHS had previously worked in local authorities and vice versa, and all had experience of cross-organisation collaboration. All stakeholders had leadership positions and a number had current or past clinical or practitioner roles.

**Table 1:** Characteristics of recruited ICSs and number of interviews completed

**ICS reference**	**Narrative summary**	**Deprivation**^a^	**Maturity**^b^	**Rural/Urban Majority**^c^	**Number of completed interviews (of stakeholders invited)**
ICS1	Stakeholders considered this ICS to be a “trailblazer” in local integrated care provision, based on work carried out over many years. Several examples of good practice were given	High	High	Urban	4 (of 5)
ICS2	Despite a high maturity rating, stakeholders felt that the ICS was at an early stage of development, with CYP service integration still an aspiration	Low	High	Rural	3 (of 4)
ICS3	Stakeholders emphasised the importance of a whole system approach. Established partnerships (non-hierarchical relationships) were noted. There were concerns that tensions may develop between local needs and national policy imperatives	Medium	High	Urban	4 (of 4)
ICS4	There had been sporadic attempts to improve integration of services in the past. There was optimism for the future, although an expectation it would require considerable resources and time to achieve system change	Medium	Medium	Rural	3 (of 5)
ICS5	Programmes of integrated care were reported, although these were not the consequence of ICS formation. Tackling health inequalities was a key focus for this ICS	High	Medium	Urban	5 (of 6)
ICS6	There was support from local authority and NHS stakeholders for greater integration. Nevertheless, stakeholders expressed scepticism, given previous poor experiences when attempting service integration	Low	Medium	Urban	3 (of 5)
ICS7	Structures for the ICS were still being developed, with support from established local NHS organisations. There were few examples of local integration of services	High	Medium	Rural	3 (of 4)

^a^Deprivation: from the Indices of Multiple Deprivation 2019 scores for the ICS footprint

^b^Maturity of the CYP system: answers to a question in an earlier online survey about perceived progress towards being a fully functional ICS for CYP, on a scale of 1–5 [low (1–2), medium (3) and high (4–5)]

^c^Rural/urban majority: whether the ICS footprint was predominantly rural or urban

Analysis of interview transcripts yielded four central themes, which are outlined below. Each overarching theme consisted of several sub-themes, as shown in the supplementary material (see Additional file 1), reflecting how the coding scheme was indexed into themes. The findings presented below include visual summaries of each theme as well as illustrative quotations. Stakeholders were assigned unique identifiers (Int1-25), which are shown alongside quotations. To ensure anonymity, no information is provided on stakeholder characteristics or linking an individual to a particular ICS.

### Theme 1: Best start in life

***“…Thinking flexibly about the whole life course and the health of the population, and it’s not just a health thing…”***

**Fig. 1:**
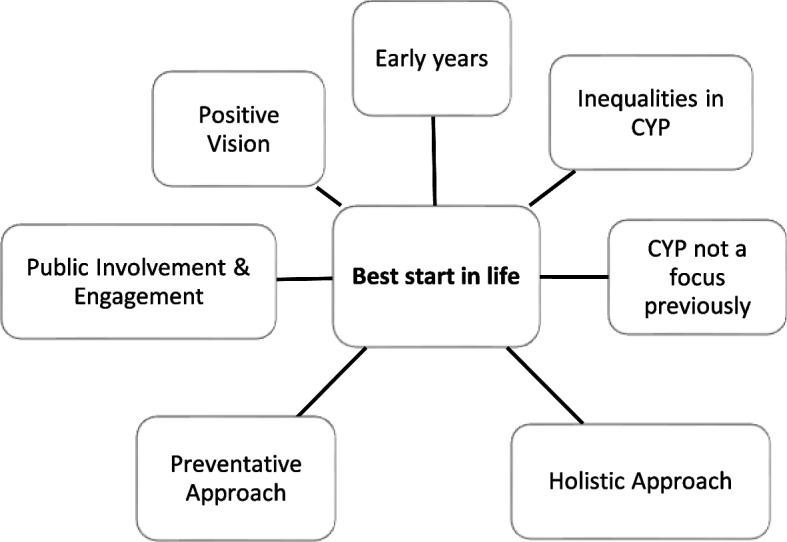
‘Best start in life’ theme

Operationally, giving CYP the ‘best start in life’ (Fig. 1) focuses on the first 1,001 days through pregnancy to the age of two, defined as the period when the building blocks for lifelong emotional, cognitive and physical development are laid down [22]. More recently introduced public health approaches highlight the importance of continuing support beyond toddlerhood. Indeed, it has been suggested that whole system support is required, with needs-driven care for CYP and families [23], a positive vision supported by stakeholders:

*“…we want all children and people to have the best start in life… And the support and health care to enable them to be safe from harm, to enjoy healthy lifestyles, to do well in learning and have skills for life.” [Int23]*

Stakeholders across the ICSs felt that prioritising CYP was key to their planning and delivery goals, while acknowledging historical barriers, where resources were directed largely towards acute service provision for adults:

*“I think it's about to start prioritising it* [children’s services]*. Up until recently, I'd say it hasn't necessarily. But I think that's due to overall government policy, I think, not having a CYP strand to it in terms of a national CYP programme sourcing down to regional ICS programmes. So, I'd say not until recently.” [Int14]*

Stakeholders shared an aspiration that the introduction of ICSs provides an opportunity to shift service priority-setting and delivery from an approach which is reactive, responding to acute, critical need, towards one which is preventative, reducing pressures on health and social care services:

“*Early help… so that children don’t have to get to the stage where they need proper medical intervention.*” *[Int12]*

It was anticipated that the latter approach would be more holistic and inclusive and encompass a range of services, such as early years support, trauma-informed care and early identification and treatment:

*“This is talking from an acute sector, but chasing community services to provide services to support families better will obviously be one area that might improve things. Ensuring that the right patients are referred into hospital. And earlier pick up and identification of problems and early management may prevent many other treatment complications later on.” [Int21]*

A holistic approach would require an organisational shift (see also: Organisational complexities), from the current situation, with resources allocated to specific organisations and budgets, to one that was focused on better CYP outcomes, resulting in efficiencies gained through earlier intervention:

*“…people then are viewing* [integration] *from their own particular viewpoints… That’s not an integrated system. The integrated system would be to say how can we design something that takes all of the cost that we are currently spending on this and intervene earlier with children in order to get a better outcome?” [Int16]*

Stakeholders from ICSs across all levels of deprivation suggested that the ICS model would enable greater attention to be given to the social determinants of health, with the ultimate aim of providing a better start in life for all CYP, recognising that tackling inequalities in outcomes, experience, and access in local areas is one of the core purposes tasked to all ICSs. Nevertheless, ICSs in more deprived areas identified the specific impact of social determinants on the health and well-being of CYP in their communities:

*“…extensive challenges around things like air quality, poverty, poor quality housing, quite significant challenges on things like family homelessness. You might see some of those societal features translating into particular patterns of demand in the health service for things like admissions for childhood asthma. There’ll be a specific range of physical health conditions, long-term conditions, that will be more pronounced… given our socioeconomic mix, than you might see in other parts of the country.” [Int6]*

Examples were given of collaborations that pre-dated the introduction of ICSs in areas of high deprivation, working across health, social care, education, and voluntary organisations to address complex, multifactorial problems that are influenced by social determinants. Bringing together all sectors working with CYP, including healthcare, social care, education, youth justice and voluntary sector was seen as an approach that could help to develop a more collaborative and interdisciplinary team of professionals:

*“The focus is about working together to really focus on improving outcomes. So, commissioning for services that really focus on prevention, early intervention, both for physical, mental, and social wellbeing, and really joining services up.” [Int25]*

A “joined-up” approach was regarded as a way to enable services to work together to more effectively meet the needs of disadvantaged CYP:

*“There are huge health inequalities within our system within the children, and poverty as being part of that, health inequalities, housing. So, I do think the integrated care agenda is probably the correct way to deal with children, looking at the whole-system approach toward the way that we deliver health and wellbeing. So, that includes schools. That includes housing. That includes activities and green spaces as well as activities for kids but also providing essential services like medical services as well as, of course, services that support, so, allied health professionals elements for children.” [Int8]*

However, there were concerns about whether ICSs would be able to address deep-rooted social determinants:

*“…It's a health-focused delivery mechanism, again, and how do we upstream and ensure effective investment and shift left, into the prevention field, which is primarily delivered through local authorities, education, third sector. So, how do we ensure that we are absolutely tackling health inequalities in that prevention space when by the time most people hit a health arena in secondary and tertiary care, we've already got established difficulties.” [Int7]*

A key subtheme was a recognition of the importance of CYP and family engagement in integrated care. Despite this, stakeholders conceded that engagement had often been fragmented, with CYP and families having only limited involvement in planning their own care, or in broader discussions about service planning. Having recognised the importance of CYP and family involvement, stakeholders highlighted that *“it’s very much a work in progress”* [Int2] that will take time and resources to ensure it works effectively:

*“It’s really, really important that we check in with parents and children and young people, because again, it’s those tests of, is what we’re planning here going to make this better for you?” [Int1]*

The involvement of seldom heard or marginalised communities was particularly limited and some stakeholders emphasised how challenging inclusion can be and that they “*haven’t got an easy answer to that*” [Int13]. However, stakeholders argued that they were now actively seeking to *“ensure that we’ve got that breadth of input”* [Int23], and saw the introduction of ICSs as a meaningful opportunity to involve CYP in decisions about their own care and to increase public engagement:

*“We’re going to be working a bit closer with our communities and engaging with them to get more of their voice through. We are very much focusing on that.” [Int15]*

### Theme 2: Local and national contexts

***“…we're really strongly linking our place priorities and interweaving it with national Long-Term Plan priorities but making sure that our delivery is nuanced...”***

**Fig. 2:**
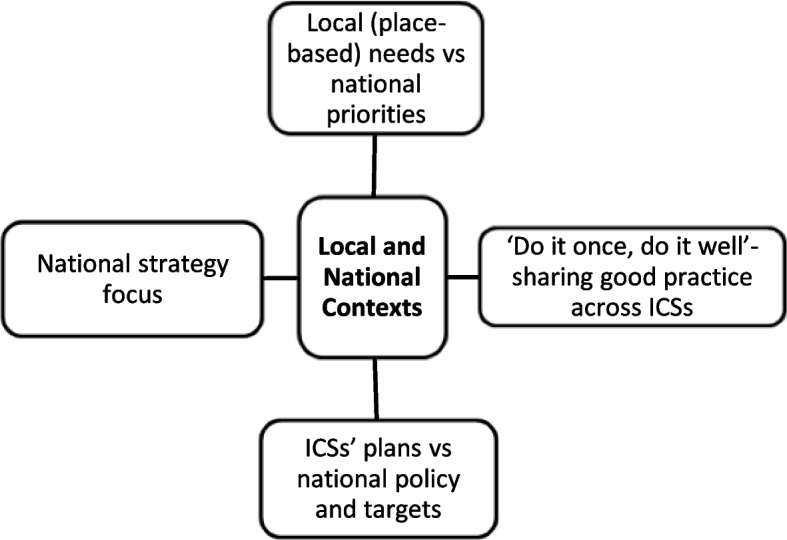
‘Local and National Contexts’ theme

ICSs were introduced within a national policy framework that sought to create opportunities to improve coordination and collaboration of local services. The interplay between national and local factors around funding, targets and priorities, mean that the subthemes underlying this theme are, necessarily, interrelated (Fig. 2).

Stakeholders discussed concerns around the ability of ICSs to meet the requirements of the NHS Long-Term Plan and the challenges of balancing national policy and targets alongside the ICS’s own local (place-based) plans and priorities, taking into consideration the “*unique characteristics*” [Int10] of the local context.

The majority of stakeholders acknowledged tensions between local and national priorities:

*“So, I’ve got a strong view that it’s not helpful to be setting detailed national programmes because it means that the attention of the NHS locally is looking up to receive that national instruction rather than looking out in terms of what would make a difference to the population we actually serve. And it ends up with a cookie-cutter model where people just implement the same thing, regardless of relative need or relative success in that implementation. But I think that’s part of the challenge that we’ve got landed with in the ICS context.” [Int6]*

This had led some ICSs to press for a greater acknowledgement of the importance of local context alongside national priorities:

*“Sometimes, what we see is that NHS England try and dictate to us how we should do things. […] And we push back and say, don’t tell us how to do it because every place’s context is different. I’m absolutely fine with agreed set of outcomes. But in every place it will be different in terms of how you deliver that because of your make up of your different organisations.” [Int16]*

Successful integration was seen to require a recognition both of local need and national policy:

*“The only right answer to that, really, is both. Definitely both. You get emerging needs in your locality and the neighbourhood that you work in. At the same time, there will be directives that come nationally that you have also got to pay attention to.” [Int19]*

For example, it was suggested that ICSs should be allowed to determine how national CYP outcomes are delivered within their local context:

*“…let’s agree what a good outcome looks like and then we can determine, and this is what we’re doing at ICS level. We’ve agreed what outcomes we’re doing for the child health and the community. They’re agreed outcomes. But how they’re going to be delivered is determined at place. Because each trust is slightly different and that’s the really positive way of doing it. You’re all aiming for the same outcomes, but you might achieve it slightly differently. I think that’s what we’re saying to NHS England. Very happy to have shared outcomes but don’t tell us how to do it.” [Int23]*

Given ICSs were in their infancy when the interviews were carried out, there was an understanding that the balance between national and local perspectives should become clearer over time:

*“we're really strongly linking our place priorities and interweaving it with national Long-Term Plan priorities but making sure that our delivery is nuanced and that it effectively addresses place identified issues.” [Int7]*

Diversity of local needs, resources, and level of service integration were acknowledged in all stakeholder interviews. This was seen as providing opportunities to learn from the experiences of others:

*“So if one area is doing really, really well, another area not doing so well with some things, how we can share that good practice and try and level up continuity. Where we can do things once, so that might be for example, commissioning services together. You know if areas have got small numbers of young people and they want to look at commissioning a service, let’s get together with all our commissioners across [the region], and look at where we can do that.” [Int9]*

## Theme 3: Funding and planning

***“There are other funding streams across the ICS, for example, in relation to ‘aging well’, but we haven’t got a ‘starting well’ fund.”***

**Fig. 3:**
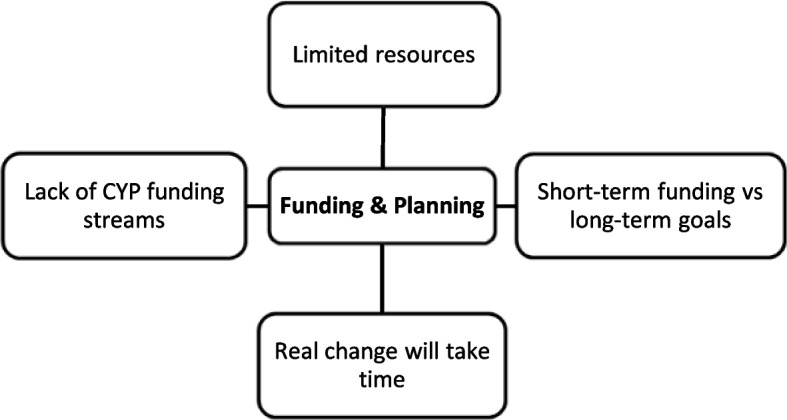
‘Funding and Planning’ theme

The development of innovative local services for CYP within existing funding and planning constraints was seen as a particular challenge for ICSs (Fig. 3). For example, while ICS stakeholders may aspire to support the “best start in life” within a population health framework (see Theme 1), current funding streams continued to focus on the provision of acute adult care:

*“You prioritise certain things through finance. And I think there's a danger that you tend to support the kind of problems in the systems, so emergency care, A&E, and all these systems where a lot of your finance goes into that.” [Int8]*

Additionally, there was a lack of resources targeted specifically at the provision of CYP services:

*“Children are quite marginalised because we tend to commission on either place-based, disease-based or processes rather than age.” [Int22]*

One stakeholder provided an example where, even after priorities were identified based on a local needs assessment, resources were not available for the CYP services required to meet that demand:

*“In our programme, we’ve prioritised based on need. We did a big piece to assess all of our outcomes data… and children’s mental health and emotional well-being is a massive issue. So, it’s a big priority for all of us both in terms of the demand curve, but also the recovery from COVID. But also, just the fact that the resource isn’t there. So even though it’s growing, we can’t keep up with the demand. That’s a big priority for us.” [Int3]*

Many national policy targets were felt to be short-term and there was an imperative to meet them, even if they contrasted with the goals of the ICS for CYP care:

*“NHS probably expects you to have outcomes in six months’ time, after ICS is in place. But in reality it’s a long-term project.” [Int25]*

Similarly, stakeholders reported that funding opportunities were often time-limited with no guarantee of future resource, or were announced at the last minute and based on national priorities, which were difficult for the new ICSs to manage as they attempted to plan for the medium and longer term:

*“But what we always get is one year, very prescribed funding and I don’t think that’s very helpful. I’m more thinking about investment in preventative and early intervention with a recognition that that won’t produce the benefit in a year. It needs to be a little bit longer term.” [Int11]*

Stakeholders reported spending much of their time working to ensure resources were in place to support the development of integrated care. One interviewee compared their daily work to a *“hamster wheel”* with a lack of capacity to have *“headspace to think about how things could be different”* *[Int2]*.

Despite the challenges of working within the existing funding and planning framework, stakeholders acknowledged that change would occur, albeit over the long-term:

*“This stuff doesn’t happen overnight. Sometimes it requires stickability. There’s something about stability of the programme as well. You can’t fly in. You really need to stick with it. So, it’s consistency and leadership, and continuity in leadership around key themes, that really do matter. But I’d say that in order to actually lead these programmes at ICS level, it needs to be a multidisciplinary team approach.” [Int2].*

### Theme 4: Organisational Complexities

***“One of the biggest barriers I come across is organisations failing to understand what other organisations do….”***

**Fig. 4:**
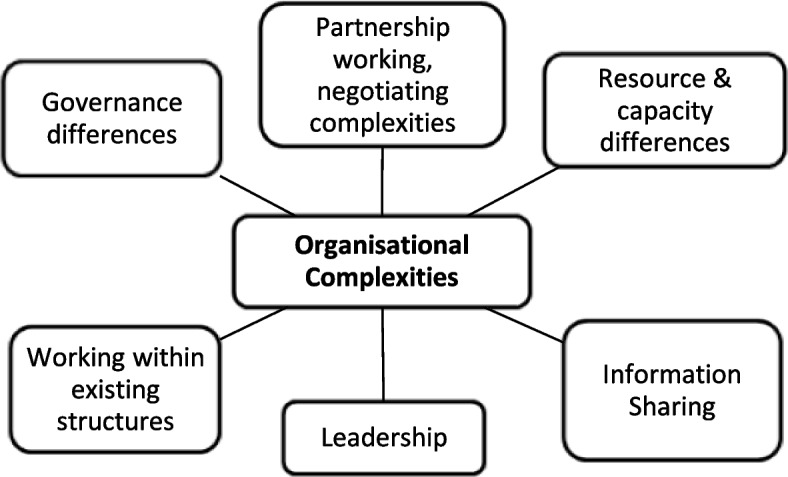
‘Organisational Complexities’ theme

The process of developing integration of CYP services requires an appreciation of organisational complexities (Fig. 4), since it involves collaboration between existing organisations and services, which have different experiences of working together and information sharing, expectations, governance arrangements, and resources and capacity:

*“There’s barriers coming out your ears, constant barriers. Workforce is a constant barrier. Resource is a constant barrier. Air time is a constant barrier, people’s day jobs. We formally funded people to be in leadership roles for the programme because that’s important. Otherwise, you lose people along the way. The system architecture changing as much as it is at the moment is a barrier because it’s creating uncertainty for so many people who are involved in and around it. They don’t know what their jobs are going to be. They don’t know what it’s all going to look like in July. That in itself is a barrier. It creates a vacuum.” [Int3]*

Siloed working, where organisations and professionals operate in isolation, was raised as a particular challenge to integration, and something that has no quick or easy solutions. Some stakeholders argued that, while there were crossovers between services, the delivery of care was still fragmented:

*“I have a phrase which is “children’s minds aren’t always quite as ordered as the services that we have designed for them”. We have quite siloed services.” [Int16]*

Stakeholders suggested potential opportunities to overcome organisational barriers to increase integration, such as “*system-wide working as part of* [a clinician’s] *role… to improve outcomes and improve that system working*” [Int9]. There was an acknowledgement of the importance of collaborative relationships that had developed over time, before the introduction of ICSs. This included partnerships with a *“really strong ethos around wanting to improve outcomes for our population”* and *“with quite a lot of passionate people that really want to do the best and look at how we can improve outcomes”* [Int9]. More recently, ICSs were actively working to develop collaborations across sectors:

*“I think that as some of the relationship work around health and local authority continues to develop...In our programme, we're working really hard at it and it's paying dividends. We've got real buy-in from some of our local authority colleagues.” [Int7]*

One stakeholder gave a specific example of a multi-disciplinary team that had been successfully created in their local area before the introduction of ICSs, with staff from a range of disciplines working together:

*“…we already have an integrated children’s system with health… we have health visitors, school nurses, midwives, family and nurse partnerships embedded in localities working in multi-agency teams with integrated leadership. It is very much a system understanding and planning approach that we have rather than working as individual organisations. So, we have had that in place for quite a few years.” [Int19]*

However, the different cultures and funding arrangements of organisations within an ICS footprint could create barriers to working together:

*“If you compare the local authority to the health, local authority is driven by a political and keeping in budget culture. Health is driven by needs-based. What the needs are of the individuals, and we put more A&E services and more elective care on, whereas the local authority has got finite budget and they have got political leadership.” [Int22]*

The challenges of building integrated services across diverse organisations led a stakeholder to suggest a collective decision-making process for distributing funds across services:

*“Ideally, if you just had one pot of money and you said, we’re going to agree collectively how we prioritise that, how much is going to go into education, how much is going to go to prevention, etc. But that’s never going to happen.” [Int25]*

Another stakeholder, while acknowledging difficulties, argued that maintaining organisations with particular specialisms was a strength of the system, with better partnership working the only realistic approach to integrating care:

*“it’s actually quite useful that we have specialists that are delivering things in different ways. We have got to work out how the links between those organisations work better then.” [Int13]*

Partnerships require leadership and effective ways of working that could bridge differences between organisational contexts. In particular, stakeholders stressed that leadership goes hand-in-hand with the development of successful partnerships, and that good leadership makes a critical difference to any organisation. A key characteristic of good leadership was someone or a group of people who would *“make everyone’s voice heard… have the voice of experts by experience…to help influence their thinking and their understanding”* and who would *“put their head above the parapet and unconditionally support* [ICS staff]*”* [Int22]. A leader should make key decisions and take responsibilities for them, and be respected across the ICS:

*“You need to have those key decision makers at the forefront of some of this work... I can’t tell you who… that might vary, I guess from ICS... in terms of who do people listen to, who has that respect within the system.” [Int18]*

By bringing together different organisations, stakeholders expected ICSs to create *“interrelationships of networks”* [Int24]. On a personal level, stakeholders suggested the importance of trust in building relationships within the ICS:

*“I think it involves the trust. It involves basic things like information sharing and a commitment to work together and a commitment to chop down the walls and to be able to problem-solve. It’s like safeguarding children, it’s that being able to have trust in the system so that you can have arguments, boundaried arguments, rather than going off in a corner and bitching about each other. I think that’s really important, and recognising the skills and the strengths and the abilities of each party and recognising that not everybody does everything. I think it’s being able to have… Good challenge is really important.” [Int24]*

Information sharing was a practical illustration of the challenges faced when working across organisations. While most stakeholders were not directly involved in systems planning, there was an awareness of the difficulties that can arise when trying to create an integrated flow of information:

*“I think that’s still sometimes challenging, because people are using different systems and they’ve got access at different levels to different systems…” [Int1]*

Nevertheless, information sharing was seen as an important component of effective integrated care:

*“It’s evidence, it’s outcomes, it’s doing it with minimum bureaucracy, it’s commitment to working in an integrated way and to sharing information, to think about how can we create an integrated system.” [Int24]*

## Discussion

The introduction of ICSs in England took place in the context of severe organisational pressures caused by the COVID-19 pandemic and with only a limited evidence-base on effective models for CYP integrated care [5]. Against this background, we carried out the first in-depth qualitative study to explore the views and experiences of professional stakeholders from a diverse sample of ICSs about the implementation and development of integrated CYP services, including plans and aspirations and the challenges faced. We identified four overarching themes from the responses elicited: 1) opportunities for a more holistic approach to health afforded by integrated care to give CYP the best start in life; 2) challenges and solutions arising from tensions between local and national contexts; 3) challenges and opportunities related to funding and planning CYP integrated care within constrained existing funding streams; 4) challenges and opportunities of integrating the work of diverse organisations.

Interviews were conducted at an early stage in the implementation of ICSs, and therefore the views of stakeholders largely reflect past and current experiences of attempting to integrate care, along with plans and aspirations for the future of ICSs. This was manifest in a common belief that CYP would be a higher priority for ICSs than they had been for health service providers historically, involving, by necessity, non-acute care provision and joint working across health, education and social care. A greater focus on CYP was seen as particularly important in the aftermath of the COVID-19 pandemic, which has had a severe impact on CYP’s mental health and wellbeing, education provision and attainment, and social care needs [24]. A shift away from the existing concentration of resources and attention towards acute medical provision would also create opportunities to embrace a longer-term, population health perspective in order to reduce inequalities through tackling the social determinants of health [7, 9, 25, 26].

There was enthusiasm among stakeholders for increased multidisciplinary and organisational collaboration in ICSs, which had been accelerated through having to adopt new ways of working during the pandemic, such as the streamlining of systems and greater use of virtual platforms. Yet challenges were also identified. Firstly, ICSs operate across existing organisations and professions, each with particular funding, statutory, governance, workforce capacity, information-sharing and leadership issues. Therefore, integrated care requires the development of mechanisms that allow disparate organisations to work together. A second concern was the limited and short-term resources available to develop integrated services, which contrasted with longer-term aspirations to develop holistic CYP services and the conviction that successful implementation of ICSs would take a number of years to complete. A related concern was the tension between meeting place-based needs through having the flexibility to develop local services versus the need to implement national policy and to meet targets which might not be appropriate for a particular ICS patch or locality.

One overarching systems-orientated concern raised by stakeholders was the extent to which integration would be possible without wholesale reorganisation of constituent services. There is a body of evidence that demonstrates the challenges of trying to develop integrated care across existing professional and organisational structures [27]. However, such major changes are likely to be disruptive and take many years to implement. The ICS model instead adopts an approach that focuses on developing and harnessing partnerships across existing organisations. Stakeholders also raised concerns about the challenges of introducing a new system during a crisis, in this case the COVID pandemic, which coincided with the national ICS roll out. Crises produce stressors and opportunities for rapid system change [28]. Such a dynamic was reported in ICSs, with the pandemic associated with multiple stressors, such as workforce exhaustion, movement of resources from CYP to adult care, and negative consequences for CYP mental health. However, new opportunities had been taken up by ICSs, including rapid adoption of virtual platforms and improved collaboration and partnership working.

### Strengths and limitations

These interviews provide a unique perspective on ICS progress during the initial phase of national roll out in England, with rich data from multiple informants across a diverse set of ICSs. The sample of ICSs was relatively small to allow for in-depth interviews to be carried out with multiple stakeholders in each ICS. Purposive sampling of ICSs ensured that selected ICSs were diverse, varying by maturity, local area deprivation and rurality, and the themes identified were widely reported across the interviews, suggesting salience for the wider body of ICSs. The interviews reflect views at a particular snapshot in time during the development of ICSs. Nevertheless, themes raised are likely to remain salient for ICSs, and a baseline against which their development can be charted. Qualitative research of this kind has particular value in the early stages of the development of ICSs before they have had an opportunity to impact CYP health outcomes, providing insights not only into existing circumstances but also plans and ambitions and how changes will be implemented over time, which can inform future research and the evaluation of progress [29, 30].

None of the recruited ICSs represented the low maturity category; reduced staff capacity due to the provision of additional services during the pandemic meant that sampled low maturity ICSs were unable to participate. However, the marker of maturity for each ICS was a rating provided in an earlier survey of ICSs, and it was clear from the interviews that despite the absence of low maturity ICSs, those included in the sample varied in their starting points, both in terms of past experiences introducing integrated services and progress towards being fully functioning ICSs for CYP. There were difficulties disentangling progress in integrated care delivery that was the consequence of the creation of ICSs from collaborations that already existed in particular local footprints, although it is likely that a clear distinction would not be realistic in most cases, where local partnerships had developed naturally over time. Stakeholders were selected for interview by the individual ICS, and so the roles represented differed between ICSs. This approach was adopted because the considerable variation between individual ICSs meant that it would be impossible to prescribe the most relevant individuals to interview. Nevertheless, ICSs were provided with guidance about the types of roles they might consider, and all stakeholders were senior members of staff who understood local CYP services. The answers provided by stakeholders represent personal perceptions around the interview topics, and we have no data on service provision or effectiveness of each ICSs. For instance, we cannot identify whether concerns about top-down, national priorities (or absence of guidance) does or does not influence services or outcomes.

Finally, a strength of the project of which this study is a part was the inclusion of public involvement and engagement activities, which helped shape the research programme. This included consultations with CYP and parents/caregivers on topic and question development for the interview guide used. The focus of the study was to canvass the views of professional stakeholders involved in rolling out ICS services, a stage at which CYP and parents/caregivers would not have experienced changes to care provision. However, CYP and parents/caregivers, particularly from seldom heard or marginalised communities, have an important voice, and their views should be elicited in future research, providing perspectives from users as well as service providers as ICS continue to develop.

## Conclusions

Internationally, progress towards CYP integrated care has been marked by variability in its conceptualisation and implementation, which often reflects the specific circumstances in which systems have been introduced [14]. Nevertheless, progress towards integrated care has become a feature of many healthcare systems, with an increased acknowledgement of the importance of the impact of social determinants on population health and the importance of involving communities in the development of services to meet their needs [31].

Maile et al. [9] in a review of the history of integrated care in England identified six lessons from past experiences. First, that integration generally delivers positive outcomes for patients, staff and the wider system; second, that divisions between organisations impede integration; third, that sustained provision of human and financial resources are required to develop integrated care; fourth, that long-term evaluation is required to support improvement and enhance credibility, which is challenging given the complexity and diversity of integrated care provision; fifth, that effective integration requires strong relationships and trust between staff groups; and sixth, that integration may be particularly effective in deprived areas because it facilitates local access to services for underserved communities. These lessons resonate with the aspirations and concerns identified from the interviews carried out with professional stakeholders during the roll-out of ICSs in England.

The stakeholders interviewed valued the opportunities provided by the introduction of ICSs to develop interdisciplinary, coordinated and joined-up care for CYP. Their views were aspirational and focused largely on plans for the future, reflecting the fact that ICSs were in their infancy and were being implemented within existing NHS and local authority structures. Even though ICSs were autonomous and were soon to be granted statutory powers, ICSs operated within frameworks, guidance and targets that often originated elsewhere, and navigating those competing demands was an important consideration for the stakeholders. Nevertheless, stakeholders had long-term ambitions to improve integration of services for CYP, taking an early years, preventative approach focused on population health and reducing health inequalities, in line with the ambitions of the NHS Long-Term Plan. This was acknowledged to be challenging within existing structures, targets and funding streams. Interviews were carried out during the first year of national ICS rollout, and changes leading to greater integration would need to take place over a number of years. Since integration is a process towards a longer-term goal, continuing evaluation will need to take place. It will be valuable to carry out a similar study when ICSs are more mature, potentially triangulating findings with other types of data from ICSs to explore the impact of integrated care on the health and wellbeing of CYP. In this way, it should be possible to use insights from real-world experiences along the path to CYP service integration to identify levers to integration and solutions to common barriers that would be of value to those seeking to introduce CYP integrated care provision in other settings.

### Supplementary Information

**Additional file 1.** ICS stakeholder interview schedule; Table of illustrative quotes for themes and subthemes.

## Data Availability

The dataset generated and analysed during the current study is not publicly available due to the risk of disclosure, but anonymised data are available from the corresponding author on reasonable request.

## Abbreviations

ICS: Integrated Care System
CYP: Children and Young People
NHS: National Health Service
