# Supplementary material for: This needs to be a journey that we’re actually on together’—the introduction of integrated care systems for children and young people in England: a qualitative study of the views of local system stakeholders during winter 2021/22
Source: BMC Health Serv Res. 2023 Dec 20;23:1448. doi: 10.1186/s12913-023-10442-6 (PMC10734058; doi:10.1186/s12913-023-10442-6)
Supplement: Supplementary file 1 — Additional file 1. ICS stakeholder interview schedule; Table of illustrative quotes for themes and subthemes. [file 12913_2023_10442_MOESM1_ESM.docx]

**‘This needs to be a journey that we’re actually on together’ - The introduction of Integrated Care Systems for Children and Young People in England: A qualitative study of the views of local system stakeholders during Winter 2021/22: Supplementary material**

- ICS stakeholder interview schedule
- Table of illustrative quotes for themes and subthemes

**ICS stakeholder interview schedule**

***Interview Schedule***

**Introduction (5 minutes)**

First of all, I would like to thank you on behalf of our research team for agreeing to take part in this research project. We are very grateful for your participation and would like you to know that your contribution is invaluable. (Succinctly describe our background and specific role within the project).

Before we start, I would like to quickly go through some organisational points. First, this interview is being recorded but I would like to assure you that the recordings are only for the purposes of the research and that we follow strict confidentiality and anonymity requirements in line with the Durham and UCL Universities research policies.

Is this all right with you?

You can choose to decline to answer any question that you do not want to answer. We are simply interested in your experiences and perspective. If there is anything you’d prefer not to say during our interview but felt it is important to record please feel free to send an email afterwards.

We estimate that the interview will take approx. 60 minutes.  Before we begin our discussion, can I just clarify whether you have any questions and if you are happy to begin?

| ***To be completed in advance and confirmed at outset of interview*** | |
| --- | --- |
| **ICS** |  |
| **Current Job** Title/Role/Team |  |
| **Time in the current post** |  |
| **Classification for interview purposes** |  |

Key:

**Bold** Questions – mandatory questions, asked to all interviewees

Bullet Questions – supplementary and optional dependent upon experiences of interviewee / flow of conversation

**Warm Up-Background details (5 minutes)**

Could you please tell me a little bit about yourself?

What is your role/background/team?

How long have you worked in this role?

**Main Body (45 minutes)**

ICS programme and priorities

1. **How do you think the ICS as a whole is prioritising the health and wellbeing of CYP? Are there any differences between overall ICS priorities and CYP priorities?**

1. **Is the development of the priorities triggered by the local need or is it a reflection of national direction.** (PIN: Try to understand differences between overall ICS priorities and the Children’s priorities – these might be different and even conflicting.)
2. **Does your ICS have a strategy, network, or programme in place for CYP services?** ---- What is it? Can you describe it? What are the overall goals/objectives of it?

- Has anything been working well? Can you provide some examples/scenarios where something has (e.g., strategy, network, or programme) worked well?

- What has not worked so well? What is hindering progress? ---- What can be done better?

1. **What do you believe are the most important CYP priorities to address to improve health and wellbeing? Can you tell me about your views on priorities such as mental health, childhood obesity and best start/readiness?**

- Has the ICS done anything yet to address these priorities? Are there any strategies or programmes in place? Can you identify any examples of good practice in relation to addressing these priorities? Can you identify any facilitators?

- Can you identify any barriers to addressing priorities and how might they be overcome?

1. **How have the priorities mentioned been addresses in relation to the existing inequalities? Why these particular priorities have been chosen?**

1. Can you perhaps quantify what additional funding you need? Do you require support to overcome any issues (e.g., cultural challenges?)

- Do these facilitators and barriers exist within the ICS? Or are they outside of the ICS?

(Consider prompting responses about key priorities such as mental health, looked after children, childhood obesity and best start/readiness with a particular focus on reducing inequalities)

1. **How do you prioritise budgets to make decisions about healthcare for CYP?**

1. How well do you feel budgets are used to treat and look after CYP?

Partnerships, resources, and capacity

1. **What does integrated care for CYP involve? What does working together for CYP look like in practice? How is it going regarding primary care, secondary health care, schools, and local authorities?**

- Has your ICS formed any partnerships to help you achieve these priorities/objectives?

1. What types of partnerships have been formed? Who’s driving the formation of partnerships? What do these partnerships entail? (PIN: ask about practicalities of working between ICSs and partnerships in terms of getting out the practical tips, strategies, and suggestion of what works well, how social and health care work with universities etc.)

1. **Have there been any challenges when forming partnerships with other services? Any resistance to change or inertia in changing habits of practice? (Note: Remember, there are a number of types of partnerships – informal, condition based, area-based, formal, systematic).**

1. Is there any support provided to develop partnerships? Who provides this support?

1. Can you tell me about the resources and capacity available within or outside your integrated care system/service to integrate care for CYP?

1. Are there any services where you think capacity (e.g., finance, staffing, space, communication) is particularly limited?

Leadership

1. **Could you describe how the leadership has been formed in your ICS?**

- Is their role influential?
- Is the leadership role paediatric driven?

1. How is the voice of clinical leaders, local authorities, schools, local NHS leaders been captured?

Information Sharing Mechanisms

1. **Can you describe how information is shared between the services that operate within your ICS?**

- **Can you tell me about the IT/digital systems in place to enable information sharing? How efficient are they?**

1. How could IT systems be more efficient and effective?

1. Do you require support to overcome issues around IT?

- Does information sharing happen formally or informally?

1. Are certain areas of the ICS better able to share information than others?

Engagement and empowerment of service users’

1. **Have you been able to include the voices/perspectives of families and CYP in the development and design of integrated care services? What does this involve?**

1. What do you do to make CYP feel welcome and understood? How are services designed to be inclusive? Does engagement reach young people who are not part of youth forums (e.g., potentially from most vulnerable groups/hard to reach), such as those in Pupil Referral Units?

(Consider prompting responses around: shared decision-making between clinicians and family members; meeting families/guardians on their own terms; flexible service delivery; respectful and open atmosphere to reduce power imbalances).

1. **Has the involvement of service users (e.g., parents/guardians, caregivers, CYP) enhanced the progress/development of integrated care services?**

1. **Have you encountered any challenges in attempting to integrate the voices of families and CYP?**

1. Do you know if your ICS has any plans for engaging service users in health care decision making and coordination of care?

Measuring integration

1. **How do you think the effectiveness of an ICS could be assessed? What would effectiveness comprise of? Any examples?**

1. How well is the national plan for ICS being implemented in your ICS? **(**Consider asking about dialogue between central government and ICSs and if ICS sees impact of their feedback to the national level if relevant)

- What do you see as key markers for measuring/assessing effective service integration?

1. Is the effectiveness of service integration measured outside clinical settings, such as schools?

1. **In terms of measuring effectiveness, does your ICS also hear from CYP as well as parents/carers?**

(Consider previous answers re: shortcomings –reiterate here) Is there anything else that could have been improved with regards to the measurement of ICS effectiveness?

COVID-19 impacts

1. **What have been the impacts of COVID-19? Please can you share your experiences, both positive and negative.**

- How has COVID-19 affected the way you work and how are you responding to it changing so quickly?

1. **Has COVID-19 inspired any changes in the environment or culture of your service/ICS?**

1. **What changes (e.g., changes in ways of working, provision of CYP services) did you anticipate you would make as a result of COVID-19? And what, if any, changes have the ICS made in practice?**
2. **What changes do you anticipate having long-term impact?**

1. **We hear a lot about how the NHS is going through difficult times, how are you helping it recover, particularly post-COVID?**

Future plans

1. **Are you aware of the ICSs future plans for integrated care for CYP? Can you describe these plans?**

Summary and close (5 minutes)

Thank you. That completes the list of questions that I have, is there anything that you would like to add or mention that you feel is important, which hasn’t been raised so far?

**Interviewer notes and comments:**

Length of interview:

Any problems with recording / any discussions off record?

Reflections (any questions not working / feedback to the team / new issues raised?)

**Table of illustrative quotes for themes and subthemes**

| **Theme 1: Best start in life** | |
| --- | --- |
| **Subthemes** | **Illustrative quotes** |
| CYP not a focus previously | “I think it's about to start prioritising it. Up until recently, I'd say it hasn't necessarily. But I think that's due to overall government policy, I think, not having a CYP strand to it in terms of a national CYP programme sourcing down to regional ICS programmes. So, I'd say not until recently.” [Int14]  “As we move into an ICS, and as we have more influence from our partners in education, and social care, I’m hoping that children will get a much higher… Get seen as being more important, because the focus of the overall system will have changed.” [Int20]  “Children get lost in the psychological fight against the tidal wave of adult need, really.  So, I think that our leaders in the ICS get the need to prioritise children.” [Int22] |
| Preventative approach | “And I would like to think with the ICS and that closer working that actually we can really work to put some of those early helps in so that children don’t have to get to the stage where they need proper medical intervention. Because that’s how it is at the moment. There is nothing for them until they’re really quite poorly, and that’s a huge shame, and we really need to work, I think, as an ICS as a priority.” [Int2]  “The focus is about working together to really focus on improving outcomes. So, commissioning for services that really focus on prevention, early intervention, both for physical, mental, and social well-being, and really joining services up. So, joining up health and care services to really support, improving outcomes particularly important in light of COVID as well where we’ve seen a significant impact both on physical and emotional well-being.” [Int25]  “You’ve got to look at how can you put as much help in as early as possible, […], thinking about how you can intervene quickly and let people help themselves but with support if you need it in the more specialist end of the market.” [Int23]  “And we’ve got lots of examples where we are where preventative health care is not being provided for children. And it’s not until they become adults… For instance, in obesity we have no services for obese children. But once they become adults, we do have services for obese adults. It just doesn’t really make sense.” [Int14]  “This is talking from an acute sector, but chasing community services to provide services to support families better will obviously be one area that might improve things. Ensuring that the right patients are referred into hospital. And earlier pick up and identification of problems and early management may prevent many other treatment complications later on.” [Int21] |
| Holistic Approach | “It needs to be thinking flexibly about the whole life course and the health of the population, and it’s not just a health thing, it’s very much education, children’s social care, voluntary sector, community, etc.” [Int24]  “One of the difficulties with children is it’s everything. Adult healthcare and health and wellbeing is split up into different bits. But children’s is everything to do with children’s health, goes all in one basket so it’s quite a broad agenda. We try and get a balance, I’m not sure we’ve got it perfectly right but it’s certainly not just responding to national agenda.” [Int17]  “… the whole life course of a child and every aspect of their lives is massively impacted on by physical and mental health, isn’t it? That’s the approach that I took.” [Int5] |
| Public Involvement and Engagement | “We’re going to be working a bit closer with our communities and engaging with them to get more of their voice through. We are very much focusing on that” [Int15]  “I think it's clear what my ambition would be… is to make co-production and co-design with children and young people the norm in [ICS].” [Int6]  “I hate the approach of let’s invite a young person to a meeting… We could always make it stronger, but we’ve definitely got the mechanisms to continue to build on and absolutely look at that engagement and involvement further.” [Int9]  “It’s how we empower our communities and how we empower our parents to come forward to work with us. That was a major part of our discussion at last weeks’ board and work going forward. We’re going to be working a bit closer with our communities and engaging with them to get more of their voice through. We are very much focusing on that” [Int15] |
| Early years | “So, I think that focus on doing a universal assessment of all of our children and young people at the right ages and stages, including 18 months which is missing at the moment. Because the earlier you can pick up that the child might have some development in an area, for whatever reason, you can actually put interventions in place that can almost eradicate that completely. So, I think that universal assessment and intervention is really important.” [Int25]  “[The programme] still emphasises the importance of the first 1,000 days, that developmental opportunity that you’ve really got to coordinate the whole of the public service around. So, I still think that early years’ model is still going to be essential with the longest-term and the most significant benefit into long-term successful lives and general health and wellbeing.” [Int6]  “I am very keen on focusing on start in life... The moment they pop out, you look at the first 1,000 days, as you will know, it’s so crucial in terms of people’s life chances and that is what we need to focus on. The trouble there is there are too many priorities, but if I had to say something I would say the ones I’ve talked about.” [Int1]  “It’s all the things linked together. Just ensuring that we are able to help with those long-term life chances in the early years.” [Int1] |
| Positive vision | “…we want all children and people to have the best start in life… And the support and health care to enable them to be safe from harm, to enjoy healthy lifestyles, to do well in learning and have skills for life.” [Int23]  “… our ambition is to close the health and wellbeing gap in outcomes for all children and young people across [name of a region removed]. No matter where they live, where they were born, where they go to school.” [Int23]  “…we've been waiting for the ICSs for a very long time, haven't we? And I think we have to be cautiously optimistic but it requires the right mature thinking around the table and I think that's the bit that we need to hold people to account for is how they really, really capitalise on this opportunity.” [Int7]  “…in children and young people’s, there is a history of collaborating across partners. That’s very much enshrined in legislation for a long, long time now. So, because we’ve got such a defined cohort, i.e., all the children and young people in [name of region removed] that creates that sense of shared vision and shared commitment that makes some of those relationships much easier.” [Int1]  “So there has to be a better way, and that has to be I think the ICSs. My hope is that by integrating this care and by breaking down those artificial boundaries... It should mean that we can provide a more seamless service for patients.” [Int21] |
| Inequalities in CYP | “There are huge health inequalities within our system within the children, and poverty as being part of that, health inequalities, housing. So, I do think the integrated care agenda is probably the correct way to deal with children, looking at the whole-system approach toward the way that we deliver health and wellbeing. So, that includes schools. That includes housing. That includes activities and green spaces as well as activities for kids but also providing essential services like medical services as well as, of course, services that support, so, allied health professionals elements for children itself.”  [Int8]    “So, when you say what is your ICS doing, we as an ICS have to address children and young people because they are such a large proportion of our population. They are also disproportionately affected by health inequalities, poverty. The fact that a lot of them are living in unsuitable housing. They disproportionately have extra long-term health conditions that if you look across the country a lot of younger people aren’t having to cope with. So, for us, we have full priority as an ICS that we have agreed going forward as part of our plans now that we have become a fully-fledged ICS.” [Int1] |
| **Theme 2: Local and national Contexts** | |
| **Subtheme** | **Illustrative quotes** |
| National strategy focus | “So, I think that there isn’t a national strategy for children. We’ve got a fantastic children’s transformation programme coming out… which I love which is the birth child stuff and also the birth childhood long-term plan. But we still don’t have a cross-party, cross-governmental big picture, what the kids mean to us, and how do we prioritise them. So we’re not bringing together. We’ve got some fantastic green shoots. We always have them in pocket of things that are big input, but we don’t tell the whole story like the Nordic countries do where they start with kids. They put kids in every policy. They prioritise the early years and hope the rest will sort itself out, is kind of the direction. We don’t work like that. We put sticking plasters over things nationally.” [Int22]  “I think one of the challenges is, is that you often have people who are brought in to purely work on the strategy, to respond to NHS England, and they’re not necessarily the people that are actually connected with the Children and Young People’s Programme as well. So, there can be a slight disconnect on occasions between feeding the information to NHS England in terms of what they need to actually hear and what’s happening in reality, and sometimes there’s a bit of a gap, actually. But there’s certainly a whole industry underway at the moment in terms of the requirements for ICS and actually meeting those, and that’s why it’s been so important to actually keep influencing about the importance of children and young people, and not missing any opportunities. And that happens sometimes. So, you’ll get people that will go out and talk to local authorities, and they won’t have any engagement with us within the Children’s Programme, and therefore children seem lower down the list of priorities. So, we’re not always as joined up as we should be as yet.” [Int9]  “I think conflicting priorities. Government, for example, the waiting list agenda is a big mandate at the moment. But it does get in the way of some of this other work that needs to happen organically from the ground. [Int1] |
| ‘Do it once, do it well’-sharing good practice across ICSs | “So, if one area is doing really, really well, another area not doing so well with some things, how we can share that good practice and try and level up continuity. Where we can do things once, so that might be for example, commissioning services together. You know if areas have got small numbers of young people and they want to look at commissioning a service, let’s get together with all our commissioners across [region], and look at where we can do that once. And also, where we can do things once, not just in terms of commissioning, but where that adds real value, where it’s a bit more cost effective.” [Int9]  ““We really try to be innovative, really try to do things where we’re taking the pressure off people in the system by doing things once together.” [Int9]  “…it’s about having pathways that make sense. It’s about providing value for money. It’s about providing good outcomes. It’s about avoiding duplication in the system, where we’re all tripping over each other because we’ve commissioned in silos.” [Int16]  “So, you might define some principles or some key elements of a pathway once, but then it would actually get delivered five times. And then, there are lots of things where actually, it absolutely, and lots of things in the children’s agenda fall into this category, it needs to be commissioned at place level to fit with local systems.” [Int16] |
| ICSs’ plans vs national policy and targets | “NHS probably expects you to have outcomes in six months’ time, after ICS is in place. But in reality, it’s a long-term project.” [Int25]  “So, we're really strongly linking our place priorities and interweaving it with national long-term plan priorities but making sure that our delivery is nuanced and that it effectively addresses place identified issues.”  [Int12]  “So, I’ve got a strong view that it’s not helpful to be setting detailed national programmes because it means that the attention of the NHS locally is looking up to receive that national instruction rather than looking out in terms of what would make a difference to the population we actually serve. And it ends up with a cookie-cutter model where people just implement the same thing, regardless of relative need or relative success in that implementation. But I think that’s part of the challenge that we’ve got landed with in the ICS context.” [Int6]  “But I do know that the legislation is being seen by some as a bit of a missed opportunity and it's a health-focused delivery mechanism, again, and how do we upstream and ensure effective investment and shift left, into the prevention field, which is primarily delivered through local authorities, education, third sector. So, how do we ensure that we are absolutely tackling health inequalities in that prevention space when by the time most people hit a health arena in secondary and tertiary care, we've already got established difficulties.” [Int7]  “We’re getting on with it. [with the national plan] Yes, I think we’ve done a lot of work to get us where we need to get. There’re some very tight timescales in that, but I think we’re going in the right direction. We’re on track at the moment.”  [Int20] |
| Local (place-based) needs vs national priorities | “We’ve had to go through the washing machine, really, with the rest of the country on a complete reinvention of the model for the Integrated Care Board and the partnership arrangements that exist across health services and wider partners then. So, it’s been more turbulent than we might have predicted because I would’ve thought that if we were looking at that set of national changes three or four years ago, we would’ve thought, we’ve already implemented that, so we can just be affirmed, can’t we? But that’s not the way that it’s been played out at all.” [Int6]  “We’ve also done a lot of work around clinical pathways, looking at… [the] hospital trusts in our area. And so, the pathways, clinical pathways, may be slightly different in those. So, you get a different service across the [hospital trusts] even if you’re in the same ICS. So, the service maybe different. We’re also trying to reduce duplication. So, what’s the sense in one hospital reviewing a pathway, and one area of our ICS reviewing a pathway and then another area doing the same thing? We can do it together and have an ICS wide pathway. So, that just helps reduce duplication of work, but also supports it being a fairer system across the whole ICS.” [Int13]  “We know what local need is, but then also, when we’re sense checking that against the long-term plan, the planning guidance that comes from NHSE every year. And we’re testing out what our local needs analysis is telling us against some of those planning frameworks. And actually, if something is ticking both boxes, it is most likely to get funded. If it only ticks one of those boxes, then it’s a more difficult conversation to have. And there are probably things that we end up funding due to national direction as opposed to local need.” [Int16] |
| **Theme 3:**  **Funding and Planning** | |
| **Subthemes** | **Illustrative quotes** |
| Limited resources | “Not in one place, but it’s who is coming forward for help and how are we as a system going to allocate resource and how are we going to use the limited resource we’ve got and do it in an evidence-based way. An example would be we have a single point of entry in one of our services where you’ve got the voluntary sector sitting there, you’ve got health, you’ve got children’s social care early help.” [Int23]  “I think one of the biggest is with regard to being on that hamster wheel of doing the day job, doing the delivery. And have you actually got the chance to actually have the headspace to think about how things could be different, and then to do that ongoing improvement work to transform those services? So, I think that there’s always that, and then there’s also that element of, how do we coproduce change directly with babies, children, young people, and their families? And how do we make sure that we’ve got the capacity and resources to actually do that really effectively? And again, to be able to go back to them and actually say, and this is the difference that you’ve actually made. So, I think we’ve still got some resourcing challenges with regard to broader healthcare engagement, improvement of tools and capacity, and also that headspace, and also that real requirement of coproduction and making that a reality consistently.” [Int9]  Developing schools’ offer in an integrated way, developing capacity in primary care. That would be my approach and that’s what we’re trying to do, but you have to turn… You can’t just stop investing in the sharp end, because there are so many people presenting in distress and in great crisis.”  [Int23]  “I think there is a challenge as always with...I think commitment is there but delivery because of workforce challenges and capacity is obviously an issue with our colleagues around that transformation space. But we know particularly around our key themes, mental health, obesity, respiratory, there is such a rise in demand when our workforce is under challenge. It's how you move people and free up enough time to move away from business as usual to thinking about transformation.”  [Int12] |
| Lack of CYP funding streams | “There are other funding streams across the ICS, for example, in relation to ‘aging well’, but we haven’t got a ‘starting well’ fund. So, that’s something that we’re having to influence considerably at the moment. It’s the funding streams that are a particular challenge, as well as exec-level support.” [Int9]  “I think one of the barriers quite often to that is there’s different funding streams for all of it. Ideally, if you just had one pot of money and you said, we’re going to agree collectively how we prioritise that, how much is going to go into education, how much is going to go to prevention, etc. But that’s never going to happen.” [Int25]  “You prioritise certain things through finance. And I think there's a danger that you tend to support the kind of problems in the systems, so emergency care, A&E, and all these systems where a lot of your finance goes into that.” [Int8]  “Unless they make these things mandatory and the funding goes with it, they’ll last for so long. And then when the council starts to run out of money, generally they start to cut the things that are not statutory, the things they don’t have to deliver. Sure Start was a great initiative. I’m not saying it’s completely gone, but it’s variable again, and that’s all down to funding. And some councils will have more money and will still fund it, and some won’t.”  [Int7]  “Children didn’t get anywhere near as much funding as adult mental health did. I don’t think children’s ever gets. And I think there’s a real gap as well. We always now start to use the expression parity of esteem between prevention and intervention, and how we make sure that there’s… The funding, there’s a bit of a left shift towards prevention as well, because a lot of the national funding coming out is for services when people have already got problems and issues and difficulties. But actually, how do we start to invest more earlier on and start to prevent some of these happening?” [Int9]  “I think sometimes what’s difficult, to speak honestly, is the funding that we get nationally, because nationally adults are generally always prioritised, aren’t they? Always baffles me a little bit because you just think well, children become adults…” [Int9] |
| Short-term funding vs long-term goals | “I worry a little bit about additional funding because it only ever comes as a one-year-type funding. Some money for asthma this year, do something with asthma. So, it’s very ring-fenced and very short term, and the difficulty then is you haven’t shifted enough, got a result of that additional money to make it sustainable going forward. And a lot of children’s work and the changes actually the financial benefits accrue years down the line. And even if it’s not years down the line they’re certainly not in year. It’s very rare you’d have an investment that would make difference in year. So, if you talk about children’s mental health, yes, if we invest early that should reduce children’s mental health services in three, four, five years’ time, not 30. If you invest early, you might also, I mean, actually that people are much healthier at 40, 50, 60. Well you’re never going to be able to calculate that benefit in that way. But what we always get is one year, very prescribed funding and I don’t think that’s very helpful. I’m more thinking about investment in preventative and early intervention with a recognition that that won’t produce the benefit in a year. It needs to be a little bit longer term.” [Int13]  “Although the money is important. But it’s about the planning for the money, and how the money comes. So, if it’s not recurrent money, you can’t set up a service for example with one year’s funding. Because then the service closes in a year, if you don’t get more funding. Or if the funding has to change to something else. So, it’s those things the way the money comes is almost more of a problem than the amount of money. So, I think that’s some of it. But I think it would be really good to have more money obviously. But I think more importantly is to know that that money is coming so you can make longer-term plans. Rather than jumping because there’s a bid coming and you’ve got until midnight on Friday to write up a bid. And it has to fit this requirement.” [Int13]  “…for all the work we’re doing, they’ll be outcome measures we will have put in place, and this is how we’re going to measure the success of that. And some of that’s long gone. You’re not going to get impact. It’s going to be medium-term outcomes.” [Int9] |
| **Theme 4:**  **Organisational Complexities** | |
| **Subthemes** | **Illustrative quotes** |
| Partnership working, negotiating complexities | “I think we view it in quite siloed ways actually. We view it in siloed ways between health and social care, but also, we don’t particularly view it from the perspective of children’s development and how children cope within families.” [Int16]  “One of the biggest barriers I come across is organisations failing to understand what other organisations do. So, everyone’s working in isolation, in their own speciality, or whatever. And they all have views on what other people should be doing. But they don’t actually understand how those people work, and how feasible...” [Int20]  “…sometimes, it feels as though Department for Education, Department of Levelling up on Communities, have also got quite key programmes of support that are relevant to children and young people. And sometimes it feels as though they’re not as mindful or as aware of the way that the ICS will work as they could be.” [Int1]  “The people, they will make their best plans in partnership with children, young people and families. There are some real principles in that, that I think need to underpin what's going to be happening at locality and neighbourhood level.” [Int6]  “… you’ve got a lot of really interested, engaged professionals. What we lacked was an executive oversight group at the ICS to actually connect us in.” [Int9] |
| Resource & capacity differences | “There are all these different ways of keeping people out of services. They are described as hard to reach. Well, it is the services that are hard to reach, not the people in some incidences.” [Int19]  “I think there’s obviously people’s capacity, isn’t there, and people’s time… There are different priorities that are coming out where we might have to pause work because the way that hospitals might be at the moment, people’s schools going back as well, but we do constantly look at the changing landscape and what work…Our role needs to be flexible, so if something happened that we needed to be picking up and pausing some of the other nonurgent work, we would do that as well.” [Int9]  “Staffing and resources is always a problem, and so I think we’re all hard pushed to keep the clinical work under wraps and on top of that. And so clearly other meetings and things come at a cost pressure. And I suppose Coronavirus and the reorganisations we’ve had to make around that have increased our workload significantly. So, I guess that’s a potential problem that we’re going to have to face.” [Int21]  “I think for program delivery clearly workforce capacity and just enough people. Workforce is a developing crisis across health and local authority, and we've known this. And even if all the money came down to deliver what we thought would be effective services actually the people, just the professionals and staff available to deliver aren't there at the moment and we know that and that's in the context of broader political issues, I think.” [Int12]  “I just think that often we operate within our bubble rather than thinking more broadly. If I was to ask a Band 5 nurse out on the ward, what’s an ICS? Are you part of an ICS? They’d look at me as though I had two heads, I’m sure. Now, as we go further along in terms of seniority, it’s likely that they’d actually have a better understanding. But I’m not sure that at grassroots practitioner level, at the moment, they’re able to see the benefits. So, what does an ICS do for me?” [Int9] |
| Information Sharing | “I think that’s still sometimes challenging, because people are using different systems and they’ve got access at different levels to different systems… And even when people are using the same system, they might have different levels of access. Like, System One, for example, I think primary care, Nought to 19, they all use System One, but they’ve got different levels of access within the system. And I do kind of get it sometimes. Even when it’s a safeguarding concern, when we know we can share information, there is something about what level of information you share… So, it’s proportionate information sharing, but proportionate is sometimes quite a challenging... It’s open to interpretation.” [Int16]  It’s evidence, it’s outcomes, it’s doing it with minimum bureaucracy, it’s commitment to working in an integrated way and to sharing information, to think about how can we create an integrated system.” [Int23]  “I think that’s still sometimes challenging, because people are using different systems and they’ve got access at different levels to different systems… And even when people are using the same system, they might have different levels of access. Like, [system name], for example, I think primary care, Nought to 19, they all use [system name], but they’ve got different levels of access within the system. And I do kind of get it sometimes. Even when it’s a safeguarding concern, when we know we can share information, there is something about what level of information you share… So, it’s proportionate information sharing, but proportionate is sometimes quite a challenging... It’s open to interpretation.” [Int16]  “As long as it’s part of a network, we don’t really care whether they’ve got NHS on their T-shirt or not. It’s evidence, it’s outcomes, it’s doing it with minimum bureaucracy, it’s commitment to working in an integrated way and to sharing information, to think about how can we create an integrated system.” [Int23] |
| Leadership | “I think because the ICS is still emerging… There’s something about using the leadership and using the expertise to actually drive that forward, there’s something about funding streams and recognising that we do need that starting well, and there’s something about building up that shared vision, so that it’s owned by the child health community. That it’s not just being imposed. This needs to be a journey that we’re actually on together.” [Int2]  “But actually, children and young people are a third of our population, between a quarter and a third of our population in the ICS depending on where you are. And actually, therefore we need to get upstream to support them because we know that 75% of mental health problems are established by the age of 18 to 25. So, we need to invest to save, and I think we need a collective leadership that understands that, and we'll have to see how that moves forward.”  [Int12]  “… enabling and allowing local authorities to fill their own leadership in this is crucial, as far as I’m concerned. If they feel like they’re being beaten over the head by NHS, and another NHS programme that comes in and thinks it knows what’s it doing, then we’ll lose it. And we won’t be able to make the effect that we need to for kids because we’re only a really small part of it. So, I think that bit about respect, and acknowledging and understanding leadership and knowledge about children, is really important.” [Int22] |
| Working within existing structures | “If you ask someone to get involved in integration because you would like to have better outcomes for children, people will go yes, that’s fine. If you get involved in integration because they say what you need to do is stop, you are sending so many EHCPs [Education, health and care plans] through. They will go no, no, no. I can’t do that, because I need to send these EHCPs through. So, it depends a bit on how you approach it in the first place and what you are trying to do, I guess. So, that’s one point. I suppose the second point is it takes a lot of time. It’s a pain in the neck. It’s quite difficult enough managing my own organisation let alone trying to then work out how I interact with other organisations and how they manage themselves. Therefore, I have to modify the way that I manage my organisation in order to deal with them. It’s all very well for me to have a conversation at a very high level and a very broad, strategic level to say let’s improve the lives of children.” [Int7]  “Again, there's still work for us to do in our programme around that primary care interface and how we get our primary care providers alongside us.” [Int12]  “So, the stuff that’s really getting traction are the bits where it’s public health, social care, and secondary care. And where we’re struggling with time and engagement, is primary care. It’s not that they don’t want to do it either, they literally haven’t got the breathing space. And I don’t think the primary care network stuff strategically is helping much because I think there’s a bit of fragmentation going on there where it should be about integration. So, yes, primary care is probably the area where I’d like to see that grow over the next year. And for that, I’d want GP leadership from across the patch to be in named leadership roles.” [Int22]  “I think it's the willingness, enthusiasm, the energy that our key leaders bring, so the co-chairs of the board.” [Int18] |
| Governance differences | “The one issue I’d say for children is, maybe a bit more for health. We’ve got our Directors of Children’s Services for social care who meet collectively and across the [location]. We don’t necessarily have the same for health because commissioners tend to be all-age commissioners rather than focusing on adults or older people or all children. Therefore, I think we’ve got a slight gap in terms of really knowing who is the strategic lead for health for children and young people. We have lots of people who work in the arena, but there’s just something about a slight gap in having that executive lead who is there to really promote the work, the strategy, the vision, and really join up with what happens around education and social care, etc.” [Int25]  ““I think across health and social care particularly, I think there is a completely different statutory framework. They have responsibilities. We have responsibilities. They are not defined in the same way. They are not talked about in the same way. So, that doesn’t help. I would say as well the firm separation within mental health and development needs I would say is probably unhelpful. I don’t think it’s unresolvable because I don’t think there is anything that is keeping us apart from as organisations. As ever, it can be slightly easier to just retreat within your own organisation at a point of difficulty rather than to work with others. It requires people to work with others.” [Int7] |
| Real change will take time | “It’s a culture change, working together. And we’re lucky in our programme that the individuals are very much invested into it. But then there is still that divide between this is health and that is social care. And it’s like no that’s not my job to do, or vice versa. Why are you looking at that? That’s social care. That’s not health. You don’t have any busy over here, or whatever it is. So, you do get that culture change. It’s going to take a long while I think. I think social services in our area are still very much saying well that’s health. The ICS is health. But it’s not. But it is. But that’s the mindset. And I just think it’s going to take some time. But, yes, there are a lot of issues with who should commission what. And I know all this stuff from previous roles as well. It’s about money. It’s also about traditional roles and having to change ways of working isn’t it.” [Int13]  “We're not going to have everything in place by then [1st April deadline]. That's unrealistic. There's that reassurance that it's going to take time...” [Int21] |
